# Environmental Tobacco Smoke During the Early Postnatal Period of Mice Interferes With Brain ^18^ F-FDG Uptake From Infancy to Early Adulthood – A Longitudinal Study

**DOI:** 10.3389/fnins.2020.00005

**Published:** 2020-01-29

**Authors:** Larissa Helena Torres, Caroline Cristiano Real, Walter Miguel Turato, Lídia Wiazowski Spelta, Ana Carolina Cardoso dos Santos Durão, Tatiana Costa Andrioli, Lorena Pozzo, Peterson Lima Squair, Marco Pistis, Daniele de Paula Faria, Tania Marcourakis

**Affiliations:** ^1^Departamento de Análises Clínicas e Toxicológicas, Faculdade de Ciências Farmacêuticas, Universidade de São Paulo, São Paulo, Brazil; ^2^Departamento de Alimentos e Medicamentos, Faculdade de Ciências Farmacêuticas, Universidade Federal de Alfenas, Alfenas, Brazil; ^3^Laboratory of Nuclear Medicine (LIM-43), Departamento de Radiologia e Oncologia, Faculdade de Medicina, Universidade de São Paulo, São Paulo, Brazil; ^4^Instituto de Pesquisas Energéticas e Nucleares, São Paulo, Brazil; ^5^Department of Biomedical Sciences and CNR Institute of Neuroscience, Faculty of Medicine and Surgery, University of Cagliari, Cagliari, Italy

**Keywords:** environmental tobacco smoke, passive smoke, neuroimaging, positron emission tomography, ^18^F-FDG uptake, glucose metabolism, longitudinal study, brain

## Abstract

Exposure to environmental tobacco smoke (ETS) is associated with high morbidity and mortality, mainly in childhood. Our aim was to evaluate the effects of postnatal ETS exposure in the brain 2-deoxy-2-[^18^F]-fluoro-D-glucose (^18^F-FDG) uptake of mice by positron emission tomography (PET) neuroimaging in a longitudinal study. C57BL/6J mice were exposed to ETS that was generated from 3R4F cigarettes from postnatal day 3 (P3) to P14. PET analyses were performed in male and female mice during infancy (P15), adolescence (P35), and adulthood (P65). We observed that ETS exposure decreased ^18^F-FDG uptake in the whole brain, both left and right hemispheres, and frontal cortex in both male and female infant mice, while female infant mice exposed to ETS showed decreased ^18^F-FDG uptake in the cerebellum. In addition, all mice showed reduced ^18^F-FDG uptake in infancy, compared to adulthood in all analyzed VOIs. In adulthood, ETS exposure during the early postnatal period decreased brain ^18^F-FDG uptake in adult male mice in the cortex, striatum, hippocampus, cingulate cortex, and thalamus when compared to control group. ETS induced an increase in ^18^F-FDG uptake in adult female mice when compared to control group in the brainstem and cingulate cortex. Moreover, male ETS-exposed animals showed decreased ^18^F-FDG uptake when compared to female ETS-exposed in the whole brain, brainstem, cortex, left amygdala, striatum, hippocampus, cingulate cortex, basal forebrain and septum, thalamus, hypothalamus, and midbrain. The present study shows that several brain regions are vulnerable to ETS exposure during the early postnatal period and these effects on ^18^F-FDG uptake are observed even a long time after the last exposure. This study corroborates our previous findings, strengthening the idea that exposure to tobacco smoke in a critical period interferes with brain development of mice from late infancy to early adulthood.

## Introduction

Exposure to environmental tobacco smoke (ETS), one of the most common indoor pollutants, is composed of both mainstream and sidestream smoke. Approximately 40% of children in the world are exposed to ETS, which is related to allergic reactions in the short-term, while it is associated to acute myocardial infarction, lung cancer, and chronic obstructive pulmonary disease in the long term (Oberg et al., 2011).

Clinical studies show that ETS leads to behavioral disorders and deleterious effects on the brain. The exposure to ETS is related to attention deficits and hyperactive behavior during childhood (Pagani, 2014), while maternal smoke during lactation causes sleep and wake disruption (Banderali et al., 2015). Also, paternal smoke in the early postnatal period of childhood has been linked with perinatal mortality, respiratory disease, neurobehavioral problems, decreased academic performance, and brain tumors (Plichart et al., 2008; Hwang et al., 2012). Adolescents exposed to tobacco smoke during prenatal period show distinct brain function in the working memory and alterations in the brain volume, especially in the cerebellum (de Zeeuw et al., 2012; Bennett et al., 2013). In rodents, exposure to mainstream smoke during a critical period of brain development leads to hyperactivity and aggressive behavior (Yochum et al., 2014), while exposure to ETS disturbs cognitive functions, synaptic proteins, and myelination process from late infancy to early adulthood (Torres et al., 2015a, b).

Positron emission tomography (PET) is a molecular imaging technique that enables studying brain function *in vivo*. The 2-deoxy-2-[^18^F]-fluoro-D-glucose (^18^F-FDG) has been widely used to evaluate changes in cerebral glucose metabolism. ^18^F-FDG is required in metabolically active tissues, and the metabolic activity of a brain region is directly proportional to the amount of ^18^F-FDG that accumulates in this region (Sokoloff et al., 1977; Welch et al., 2013).

Relatively few studies evaluated the effects of tobacco smoke on brain glucose metabolism by PET imaging and focuses on dependence by nicotine in humans. In a context of tobacco craving and exposure to cues that are related to tobacco, heavy smokers showed rise in glucose metabolism in the anterior cingulate gyrus, orbitofrontal cortex, dorsolateral prefrontal cortex, anterior insula, and sensorimotor cortex (Brody et al., 2002). In addition, smokers treated with bupropion, a norepinephrine and dopamine reuptake inhibitor, showed decrease in glucose metabolism in the anterior cingulate cortex (Brody et al., 2004). Costello et al. (2010) reported that bupropion and practical group counseling reduce glucose metabolism in the posterior cingulate gyrus, with association between cigarette use and ^18^F-FDG uptake in the occipital gyrus and parietal–temporal junction (Costello et al., 2010). However, there is still a lack of studies evaluating the effects of tobacco smoke on glucose metabolism during the brain development period. Thus, our aim was to investigate the effects of ETS during the early postnatal period on glucose metabolism in a longitudinal preclinical study, by ^18^F-FDG PET imaging during mice infancy, adolescence, and adulthood.

## Materials and Methods

### Animals

C57BL/6 mice were obtained from the animal facility of the School of Medicine of University of São Paulo and were housed at 20–22°C with a 12 h/12 h light/dark cycle with water and commercial pellet food for small rodents from Nuvital (Nuvilab CR-1; Colombo, Brazil) *ad libitum*. All of the procedures were approved by the Ethics Committee of the School of Medicine (027/14) and the School of Pharmaceutical Sciences (P446/14), University of São Paulo.

### Experimental Design

The size of each litter was randomly adjusted to six to seven pups within the first day after delivery, as previously described by Torres et al. (2015b). The C57BL/6 pups were exposed to ETS as described by Lobo-Torres et al. (2012). Briefly, the pups, together with their mothers, were subjected to two exposure sessions per day of 1-h each (1 h at 8 a.m. and 1 h at 5 p.m.) to a mixture of mainstream and sidestream tobacco smoke from reference cigarettes 3R4F (College of Agriculture, University of Kentucky). The exposure was performed from the 3rd (P3) to the 14th (P14) days of life, within a chamber measuring 564 × 385 × 371 mm. The levels of CO in the chamber during the exposure (470.2 ± 90.93 ppm) and measurements of the exposure biomarkers (COHb: 21.62 ± 1.80%; plasma nicotine: 139.94 ± 13.02 ng/mL; plasma cotinine: 113.65 ± 16.78 ng/mL) were similar to previous studies from our group (Torres et al., 2015a, b). Control subjects were exposed to the same experimental conditions but inhaled compressed air only.

Based on Vanhove et al. (2015), the number of animals required for imaging studies for changes about 20–25% is four to six animals. Thus, in the present study, we opted to use five animals of each sex in each group. Nonetheless, we used the isogenic C57Bl/6 mice in order to reduce intra-animal variability.

After the exposure period, 19 animals were used from P15 to P65 to evaluate the regional brain metabolism of the animals with ^18^F-FDG on PET/CT during infancy (P15; *n* = 5 females ETS-exposed and *n* = 4 females control; *n* = 5 males ETS-exposed and *n* = 5 males control), adolescence (P35; *n* = 4 females ETS-exposed and *n* = 4 females control; *n* = 5 males ETS-exposed and *n* = 5 males control), and adulthood (P65; *n* = 5 females ETS-exposed and *n* = 4 females control; *n* = 5 males ETS-exposed and *n* = 5 males control) in a longitudinal study.

### ^18^F-FDG-PET/CT Imaging

Positron emission tomography/CT images were acquired using a protocol modified from Welch et al. (2013). Briefly, animals received about 19 MBq (18.86 ± 2.70) of ^18^F-FDG intraperitoneally (i.p.). After 65 (67 ± 5) min of injection (biodistribution period of the radiotracer), animals were anesthetized with isoflurane (2% in O_2_) and positioned in an equipment bed with the brain in the center of the field of view (FOV). The scanner used was an Albira PET-SPECT-CT (Bruker Biospin, Valencia, Spain), for small animals. The static PET image was acquired for 50 min with 94.4 mm of trans-axial FOV. A CT scan was acquired immediately after, with 400 projections, 45 kVp, and 400 μA and magnification factor of 1.46. After acquisitions, PET images were reconstructed using maximum-likelihood expectation–maximization (MLEM), with 12 iterations, and corrected for radioactive decay, scatter, and random, but not for attenuation. CT was reconstructed using filtered back projection (FBP) algorithm.

### PET Image Analysis

Positron emission tomography image analysis was performed with PMOD 3.4 software (PMOD^TM^ Technologies Ltd., Switzerland). The scans were manually co-registered to: (1) own animal CT for analysis in the different animals’ age and (2) to a T2 weighted MRI template (available in the PMOD software) in the adult animals’ analysis to facilitate the identification of different brain regions.

In the analysis of the brain in the different ages, manual volumes of interest (VOIs) were drawn in the PET images fused to the CT (Zovein et al., 2004). Due to the small size of the infant animals’ brain, to allow comparison with adolescent and adult mice, the VOIs for all ages were defined as whole brain, left and right brain hemispheres, frontal cortex, and cerebellum, always using the skull defined by the CT as a border line. When analysis was restricted to adult animals, PET image was co-registered to the MRI template and different brain regions considered in the analysis (whole brain, brainstem, cortex, cerebellum, left and right amygdala left and right striatum, left and right hippocampus, cingulate cortex, basal forebrain and septum, thalamus, hypothalamus, and left and right midbrain).

The ^18^F-FDG uptake is presented as a standardized uptake value (SUV) which is calculated as radioactivity concentration (kBq/cc) divided by the ratio between injected dose (kBq) and animal body weight (g).

### Statistical Analysis

**FIGURE 1:**
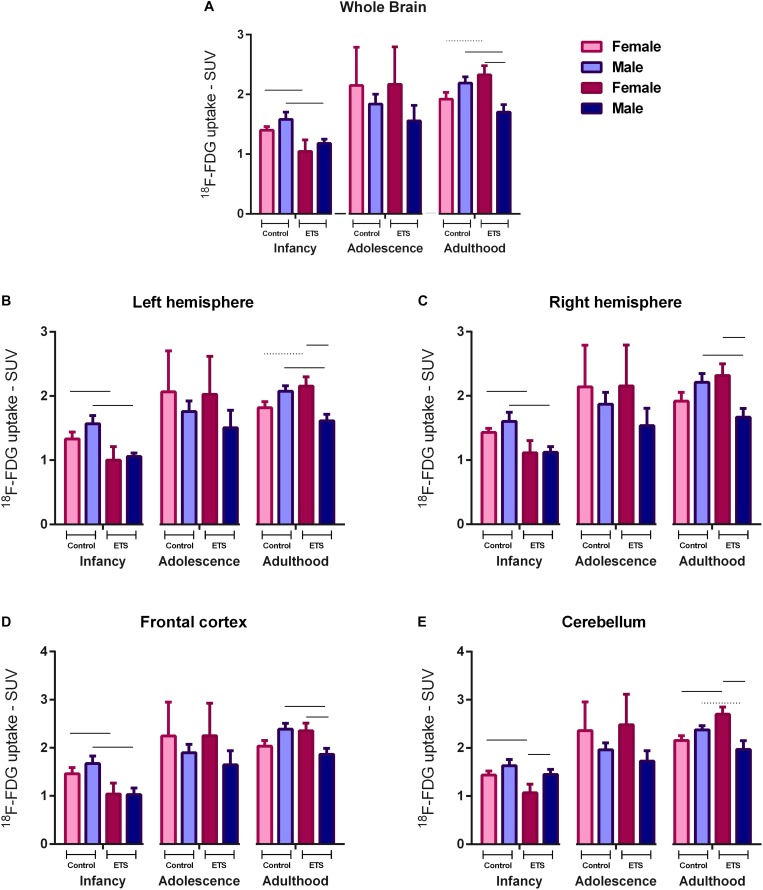
^18^F-FDG uptake in the whole brain **(A)**, left hemisphere **(B)**, right hemisphere **(C)**, frontal cortex **(D)**, and cerebellum **(E)** for female and male infant (*n* = 5 females ETS-exposed and *n* = 4 females control; *n* = 5 males ETS-exposed and *n* = 5 males control), adolescent (*n* = 4 females ETS-exposed and *n* = 4 females control; *n* = 5 males ETS-exposed and *n* = 5 males control), and adult (*n* = 5 females ETS-exposed and *n* = 4 females control; *n* = 5 males ETS-exposed and *n* = 5 males control) mice exposed to ETS during the early postnatal period. Three-way mixed ANOVA with repeated measures (groups × VOIs) and *post hoc* paired *t*-test (Bonferroni) with multiple comparison correction. *Continuous bar: p* < 0.05. *Dashed bar:* trend toward statistical significance.

As in young animals it is difficult to analyze small brain areas, due to the limited PET imaging spatial resolution, and in order to compare infancy, adolescence, and adulthood, we analyzed brain glucose metabolism in the following brain areas: whole brain, left and right brain hemispheres, frontal cortex, and cerebellum. Thus, we performed a three-way mixed ANOVA with repeated measures, considering groups as between and time and VOIs (whole brain, frontal cortex, cerebellum, right hemisphere, and left hemisphere) in infancy, adolescence, and adulthood as within-subject factors. Bonferroni *post hoc* test with multiple comparison correction was performed to test ^18^F-FDG uptake differences between the time points and groups for each VOI (Figure 1). PET imaging of adult animals was analyzed by a two-way ANOVA, considering groups as between and VOIs (whole brain, brainstem, cortex, cerebellum, amygdala, striatum, hippocampus, cingulate cortex, basal forebrain and septum, thalamus, hypothalamus, and midbrain) as within-subject factors. Bonferroni *post hoc* with multiple comparison correction was performed to test ^18^F-FDG uptake differences between the groups for each VOI (Figure 3). The data were analyzed using SPSS Statistics 20 Software, Armonk, NY: IBM Corp., United States and data were plotted using GraphPad Prism 6 Software, La Jolla, CA, United States. Results are presented as mean ± standard error. Differences with a probability of 95% (*p* < 0.05) were considered statistically significant.

## Results

### ETS During the Early Postnatal Period Decreased Brain ^18^F-FDG Uptake in Infant Mice

Positron emission tomography scan data of glucose uptake for male and female infant, adolescent, and adult mice exposed to ETS during the early postnatal period were analyzed by a three-way mixed ANOVA with repeated measures (groups × VOIs) and Bonferroni *post hoc* test with multiple comparison correction. We found a significant effect for the factors VOIs (*F*_4_,_56_ = 33.7333; *p* < 0.00010) and time (*F*_2_,_8_ = 10.99; *p* < 0.0001), and a significant VOIs × time interaction (*F*_8_,_112_ = 4.592, *p* < 0.0001). We also had a significant VOIs × time × group interaction (*F*_24_,_112_ = 1.753; *p* < 0.05).

**FIGURE 2:**
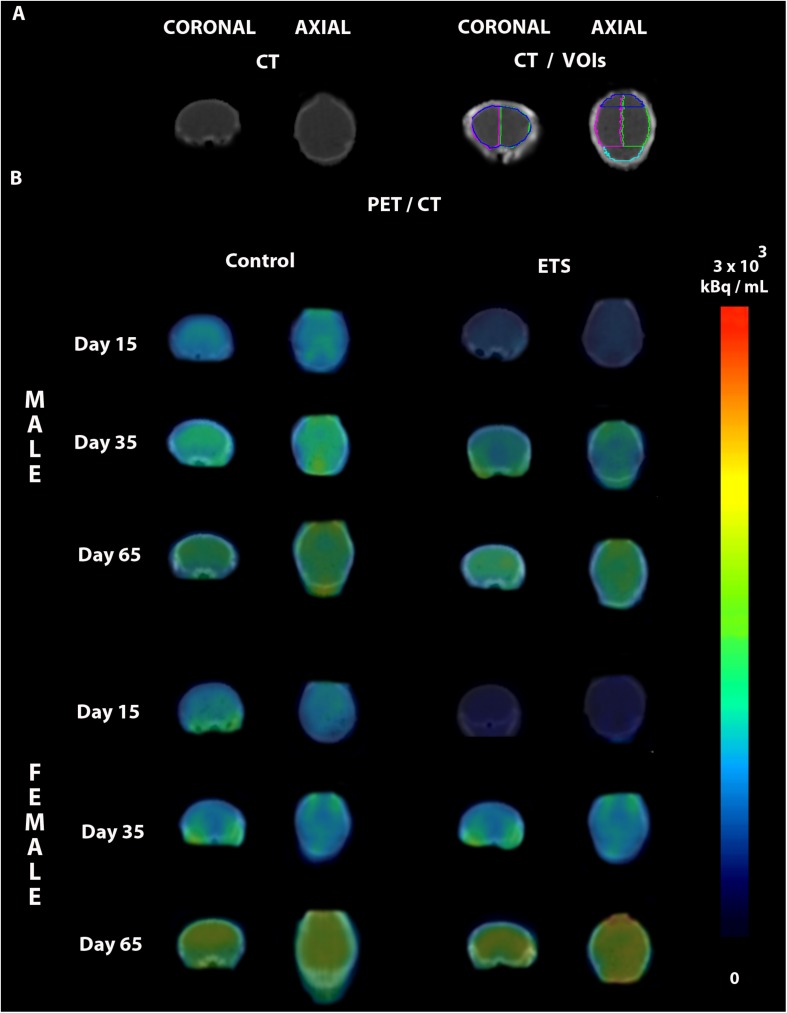
Representative PET/CT brain images of male and female mice exposed to ETS during the early postnatal period and the control group. **(A)** CT images in different anatomical planes in the top left, and CT with the drawn volumes of interest (VOIs) used for quantification in the top right (whole brain, left hemisphere, right hemisphere, frontal cortex, and cerebellum). **(B)** PET images fused to the CT images of infant (Day 15), adolescent (Day 35), and adult (Day 65) male and female mice of the control and ETS groups.

The *post hoc* analysis revealed that all mice showed reduced ^18^F-FDG uptake in infancy, compared to adulthood in all analyzed VOIs. Regarding the females of ETS group, the ^18^F-FDG uptake was also lower during infancy when compared to adolescence. As detailed at Table 1, in infancy, both male and female mice ETS-exposed had lower ^18^F-FDG uptake in the whole brain, left and right hemisphere and frontal cortex, compared to the control group. Females showed decreased ^18^F-FDG uptake in the cerebellum (Figure 1 and Table 1). When mice reached adulthood, males showed a reduction in ^18^F-FDG uptake when compared to controls in all VOIs analyzed (Figure 1 and Table 1). However, in the females a different pattern occurred, as they had higher ^18^F-FDG uptake in the whole brain, left hemisphere, and cerebellum, when compared to the control group (Figure 1 and Table 1). When both ETS-exposed groups were compared, the *post hoc* analysis showed a reduction in ^18^F-FDG uptake in the males in all the VOIs evaluated when compared to females (Figure 1 and Table 1). Figure 2 shows a representative brain PET/CT scans of mice exposed to ETS during the early postnatal period and the control group during infancy, adolescence, and adulthood.

**TABLE 1:** Detailed description of the statistical analysis of ^18^F-FDG uptake in infancy, adolescence, and adulthood mice in distinct brain regions.

**VOI**	**^18^F-FDG uptake**		
	**ETS-exposed**	**ETS-exposed vs. control**	
	**Male vs. female**	**Male**	**Female**
**Infancy**			
Whole brain	ns	↓ *p* = 0.020	↓ *p* = 0.013
Left hemisphere	ns	↓ *p* = 0.010	↓ *p* = 0.026
Right hemisphere	ns	↓ *p* = 0.013	↓ *p* = 0.036
Frontal cortex	ns	↓ *p* = 0.010	↓ *p* = 0.031
Cerebellum	↓ *p* = 0.014	ns	↓ *p* = 0.021
**Adolescence**			
Whole brain	ns	ns	ns
Left hemisphere	ns	ns	ns
Right hemisphere	ns	ns	ns
Frontal cortex	ns	ns	ns
Cerebellum	ns	ns	ns
**Adulthood**			
Whole brain	↓ *p* = 0.060	↓ *p* = 0.019	↑ *p* = 0.068
Left hemisphere	↓ *p* = 0.060	↓ *p* = 0.011	↑ *p* = 0.072
Right hemisphere	↓ *p* = 0.015	↓ *p* = 0.024	ns
Frontal cortex	↓ *p* = 0.026	↓ *p* = 0.015	ns
Cerebellum	↓ *p* = 0.040	↓ *p* = 0.062	↑ *p* = 0.029

*Each *p*-value is adjusted for multiple comparisons. ↓, decreased ^18^F-FDG uptake; ↑, increased ^18^F-FDG uptake.*

### ETS During the Early Postnatal Period Decreased ^18^F-FDG Uptake in Adult Male Mice in Distinct Brain Regions

Positron emission tomography scan data of glucose uptake for male and female adult mice exposed to ETS during the early postnatal period were analyzed by two-way ANOVA (VOIs × treatment) with Bonferroni *post hoc* test with *p*-values corrected for multiplicity. We found a significant effect for VOIs (*F*_15_,_240_ = 7.101, *p* < 0.0001) and treatment (*F*_3_,_240_ = 59.5, *p* < 0.0001) factors; however, the interaction between them was not significant (*F*_45_,_240_ = 0.033, *p* > 0.999; see Table 2 for detailed description of the statistical analysis).

**TABLE 2:** Detailed description of the statistical analysis of ^18^F-FDG uptake in adult mice in distinct brain regions.

**VOI**	**^18^F-FDG uptake**		
	**ETS-exposed**	**ETS-exposed vs. control**	
	**Male vs. female**	**Male**	**Female**
Whole brain	↓ *p* = 0.033	ns	ns
Brainstem	↓ *p* = 0.020	ns	↑ *p* = 0.021
Cortex	↓ *p* = 0.058	↓ *p* = 0.058	ns
Cerebellum	ns	ns	ns
Left amygdala	↓ *p* = 0.012	ns	ns
Right amygdala	ns	ns	ns
Left striatum	↓ *p* = 0.003	↓ *p* = 0.038	ns
Right striatum	↓ *p* = 0.019	↓ *p* = 0.033	ns
Left hippocampus	↓ *p* = 0.001	↓ *p* = 0.024	ns
Right hippocampus	↓ *p* = 0.008	↓ *p* = 0.053	ns
Cingulate cortex	↓ *p* = 0.0003	↓ *p* = 0.028	↑ *p* = 0.069
Basal forebrain and spetum	↓ *p* = 0.049	ns	ns
Thalamus	↓ *p* = 0.0007	↓ *p* = 0.022	ns
Hypothalamus	↓ *p* = 0.033	ns	ns
Left midbrain	↓ *p* = 0.0008	ns	ns
Right midbrain	↓ *p* = 0.0007	ns	ns

*Each *p*-value is adjusted for multiple comparisons. ↓, decreased ^18^F-FDG uptake; ↑, increased ^18^F-FDG uptake.*

**FIGURE 3:**
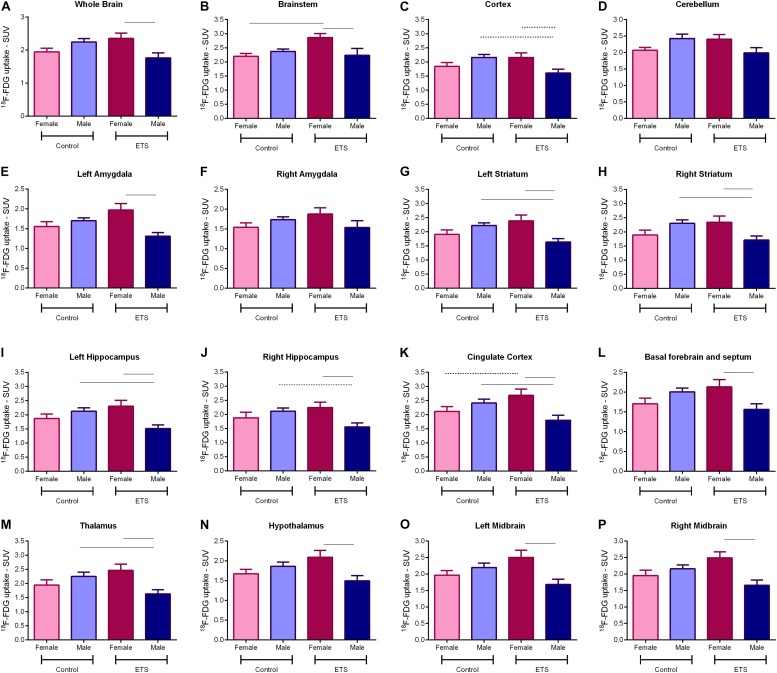
^18^F-FDG uptake for adult mice (*n* = 5 females ETS-exposed and *n* = 4 females control; *n* = 5 males ETS-exposed and *n* = 5 males control) in the whole brain **(A)**, brainstem **(B)**, cortex **(C)**, cerebellum **(D)**, left amygdala **(E)**, right amygdala **(F)**, left striatum **(G)**, right striatum **(H)**, left hippocampus **(I)**, right hippocampus **(J)**, cingulate cortex **(K)**, basal forebrain and septum **(L)**, thalamus **(M)**, hypothalamus **(N)**, left midbrain **(O)**, and right midbrain **(P)**. Two-way ANOVA with Bonferroni *post hoc* test and adjusted *p*-values for multiple comparisons. *Continuous bar: p* < 0.05. *Dashed bar:* trend toward statistical significance.

**FIGURE 4:**
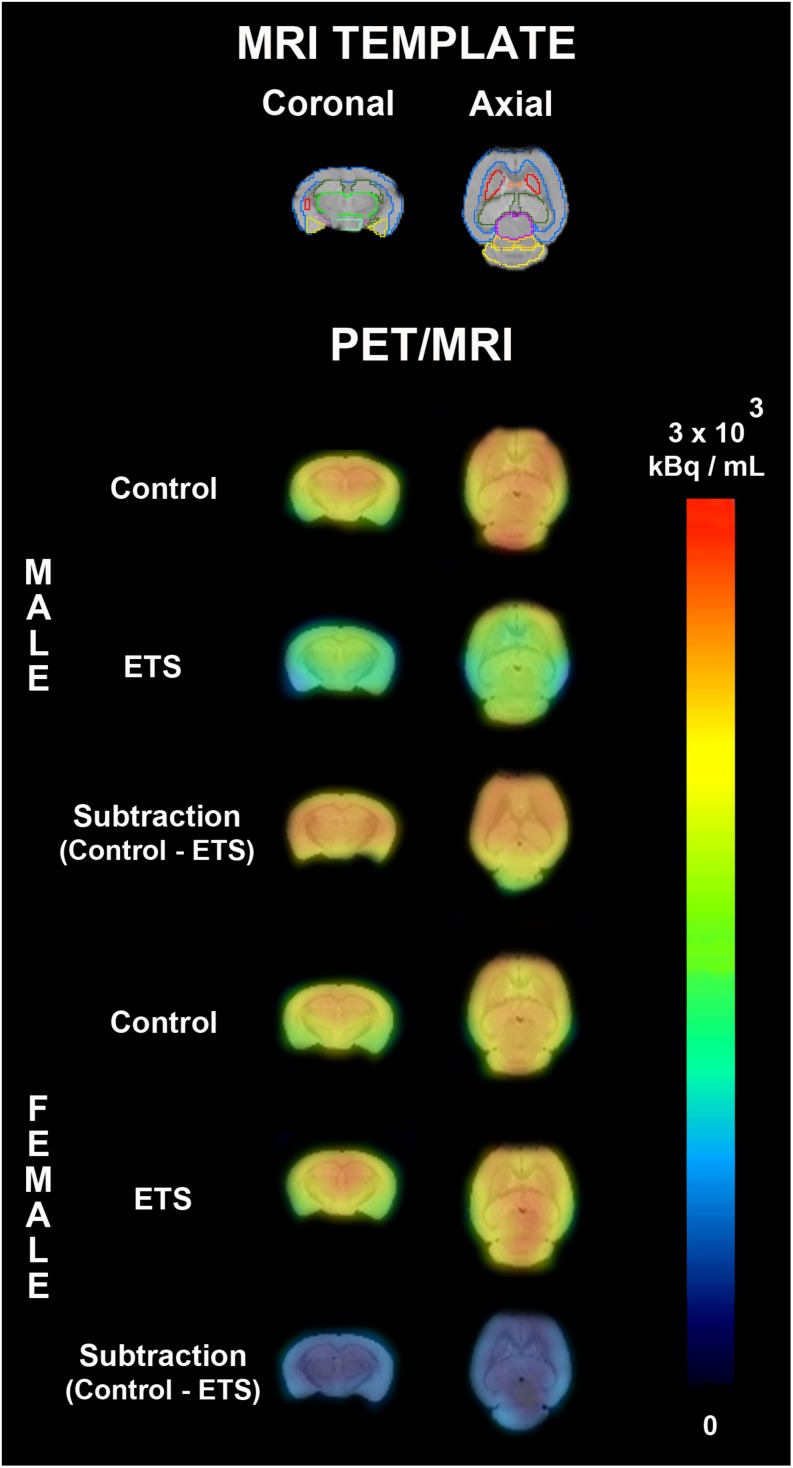
PET average images of female and male adult mice exposed to ETS during the early postnatal period and the control group. On the top, a MRI template used for drawing volumes of interest (whole brain, brainstem, cortex, cerebellum, left amygdala, right amygdala, left striatum, right striatum, left hippocampus, right hippocampus, cingulate cortex, basal forebrain and septum, thalamus, hypothalamus, left midbrain, and right midbrain). The first two rows represent PET average images fused to MRI template and the third row represent the difference of PET average image fused to MRI template between control group and ETS exposed mice.

The *post hoc* analysis showed that exposure to ETS during the early postnatal period decreased ^18^F-FDG uptake in adult male mice when compared with adult female mice in the whole brain, brainstem, left amygdala, left and right striatum, left and right hippocampus, cingulate cortex, basal forebrain and septum, thalamus, hypothalamus, and left and right midbrain. There was a trend of statistical significance in the cortex (Figure 3 and Table 2). We also observed that adult male mice exposed to ETS showed a decrease in glucose metabolism when compared with male mice from the control group in the left and right striatum, left hippocampus, cingulate cortex, and thalamus (Figure 3 and Table 2). It was also detected a trend toward statistical significance in the cortex, and right hippocampus (Figure 3 and Table 2). Regarding adult female mice exposed to ETS, we observed an increase in glucose uptake when compared with female mice from the control group in the brainstem, with trend toward statistical significance in the cingulate cortex. Figure 4 shows brain PET/CT average scans of female and male adult mice exposed to ETS during the early postnatal period and the control group.

## Discussion

To the best of our knowledge, this is the first study that investigated the effects of ETS exposure during brain development on ^18^F-FDG uptake in the brain of mice. By PET imaging, we observed that ETS exposure during the early postnatal period decreased brain ^18^F-FDG uptake in both male and female infant mice and in adult male mice in distinct brain regions and increased ^18^F-FDG uptake in adult female mice in the brainstem and cingulate cortex. In addition, male ETS-exposed mice showed decreased ^18^F-FDG uptake when compared to female ETS-exposed. These results are in accordance with previous studies of our group and with studies that show that exposure to tobacco smoke during brain development can affect the central nervous system. Exposure to tobacco smoke extract during gestational period of Sprague–Dawley rats decreased nicotinic and serotonin receptors in different brain regions (Slotkin et al., 2017). In addition, postnatal exposure to tobacco smoke leads to impairment in the myelination, learning, and memory, and induces oxidative stress and lower brain-derived neurotrophic factor (BDNF) and synaptic proteins levels (Stangherlin et al., 2009; Lobo-Torres et al., 2012; Torres et al., 2015a, b).

The exposure biomarkers of the present study were similar to Obot et al. (2004), Amos-Kroohs et al. (2013), and Torres et al. (2015a, b, 2019a, 2019b). Nwosu and Kum-Nji (2018) suggested that the classification as passive or active smoking could be done according to serum cotinine levels. Thus, serum cotinine levels between 0.05 and 10 ng/mL can be considered as passive smokers and >10 ng/mL as active smoking. Although cotinine concentration of the present study could be considered as active smoker by Nwosu and Kum-Nji (2018), the classification was based in children and adolescent under 17 years old and not in rodents. Moreover, the authors did not mention how long after tobacco smoke exposure the blood was collected. In the present study, due to the weaker binding affinity of CO for mouse hemoglobin when compared to that of human hemoglobin (Watson et al., 1987), blood collection for the quantification of the biological markers was performed immediately after the ETS exposure. Thus, the cotinine levels data reflect the peak of cotinine, as the half-life of plasma nicotine in rodents is 0.9–1.1 h.

Neuronal activity requires high energy, mainly in the level of synaptic connections and signaling transduction pathways (Sokoloff, 1999). In this scenario, ^18^F-FDG brain uptake correlates with brain metabolic activity, since we can predict brain function through the relationship between energy consumption and neuronal activity (Shulman et al., 2004). Small animal PET imaging in longitudinal studies allows *in vivo* quantification of brain metabolic activity in the same animal during different periods of life making possible to analyze how brain behave in different stages and how xenobiotics might be able to interfere in the homeostatic state. Our data showed decreased ^18^F-FDG uptake in infancy, suggesting that ETS exposure is affecting brain neuronal activity during this important period of brain development. In fact, it is known that in humans, childhood is a period in which the central nervous system is under active development, maturation, and with open critical periods of synaptic plasticity, with the higher levels of metabolism, reaching a peak on the fourth year of life (Chugani and Phelps, 1991). Until the ninth year of life there is a plateau, followed by a steady decline until adulthood, in the second decade of life, when the prefrontal cortex has completed its maturation (Kennedy and Sokoloff, 1957). During normal aging, brain passes through structural and function changes in white and gray matters, reflecting in a declined metabolic activity (Moeller et al., 1996).

It is interesting to note that even a long time after the last exposure, adult mice showed changes in ^18^F-FDG uptake in distinct brain regions, which were sex dependent. Adult male mice exposed to ETS during brain development showed decreased ^18^F-FDG uptake compared with adult female mice or with male controls in different brain regions. In fact, sex seems to be an important factor in brain response to ETS. A clinical study showed that nicotine administered by patch induced increased brain ^18^F-FDG uptake in females than males during a Continuous Performance Task or the Bushman Competition and Retaliation Task tests (Fallon et al., 2005). A previous study of our group also showed that the effects of ETS exposure during the early postnatal period are sex-dependent, as infant female mice showed poorer performance in learning and memory tests than males (Torres et al., 2015b).

Garcia et al. (2013) revealed that sex is determinant for post-hypoxic depression and recovery. Persistent post-hypoxic depression is more recurrent in male mice than female and glucose supplementation improves post-hypoxic recovery rhythmogenesis only in female mice (Garcia et al., 2013). These data are relevant, since post-hypoxic depression is involved in the pathogenesis of sudden infant death syndrome (SIDS). About 60% of the children affected by SIDS are male (Richardson et al., 2010). The exposure to ETS is related to higher risk of SIDS, a syndrome that has no known mechanism, but it requires immature cardiorespiratory control and impairments in sleep arousal (Mitchell and Milerad, 2006; Moon et al., 2016). The brainstem is associated with respiratory and cardiovascular responses, therefore is related to pathogenesis of SIDS and it is susceptible to ETS exposure. Previous studies showed that exposure to ETS in the early postnatal period decreased myelin-specific proteins and alter receptors and enzymes of the endocannabinoid system in the brainstem (Torres et al., 2015a, 2019a). The present study corroborates these findings since we observed that ETS exposure decreased ^18^F-FDG uptake in ETS-exposed male mice compared with female mice and increased ^18^F-FDG uptake in ETS-exposed female mice compared with control group.

Environmental tobacco smoke exposure during the early postnatal period induced a similar result in the left amygdala, which have a key role in the acquisition of memory related to fear conditioning (Fujisaki et al., 2004). Amygdala is associated to emotional experience, including fear and anxiety (De Bellis et al., 2000; de Oliveira et al., 2013). In fact, pediatric generalized anxiety disorder was associated to higher amygdala volumes (De Bellis et al., 2000). Active smokers have significantly reduced amygdala volumes compared with non-smokers (Luhar et al., 2013). In line with these results, preclinical studies have shown that the exposure to ETS during postnatal periods leads to anxiety-like behavior in a short- and long-term withdrawal (Abreu-Villaça et al., 2015; de la Peña et al., 2016; Torres et al., 2019b). Taken together, these data suggest that the anxiety behavioral disorders related to tobacco smoke may be associated with amygdala alterations.

In the present study, we observed that exposure to ETS decreased ^18^F-FDG uptake in striatum of adult male mice. This data are consistent with studies that revealed that ETS exposure during a critical period induced oxidative stress, decreased synaptic proteins levels and BDNF, and modified elements of the endocannabinoid system in the striatum (Lobo-Torres et al., 2012; Torres et al., 2019a, b). The striatum, constituted by the caudate nucleus and putamen, is involved in motor, cognitive, and limbic functions, and is involved in the neurobiology of addiction (Burton et al., 2015). Addictive drugs increase dopamine levels in mesolimbic system, especially in the dorsal and ventral striatum/nucleus accumbens (Hyman et al., 2006). Romoli et al. (2019) reported that exposure to nicotine during the early postnatal period increased nicotine consumption during adulthood, effect mediated by dopaminergic neurons in the midbrain, that contains dopaminergic neurons that are located in the ventral tegmental area and in the substantia nigra, regions that are important for the development of drug addiction (Björklund and Dunnett, 2007; Romoli et al., 2019).

Weinstein et al. (2010) observed that smokers showed increased craving scores after watching a videotape with smoking scenes, which were associated with brain ^18^F-FDG uptake in the ventral striatum, anterior cingulate, orbitofrontal cortex, middle temporal lobe, hippocampus, insula, midbrain, and thalamus (Weinstein et al., 2010). In addition, Domino et al. (2000) showed that nicotine increases regional cerebral blood flow, evaluated by PET, in the thalamus, pons, primary visual cortex, and cerebellum of tobacco smokers. These individuals also showed reduction in the hippocampal area (Domino et al., 2000; Hanlon et al., 2014). We observed that ETS decreased glucose metabolism in ETS-exposed male mice compared with female mice and with control group in the hippocampus, one of the main areas that is involved in learning and memory. Indeed, ETS exposure during the early postnatal period decreased synaptic proteins and BDNF in hippocampus and induced impairment in the learning and memory from late infancy to early adulthood (Torres et al., 2015b).

It is important to point out the limitations of the present protocol. In order to evaluate the long-lasting effect of tobacco smoke exposure during the early postnatal period, we used a longitudinal study to measure ^18^F-FDG uptake from infancy to adulthood. Although longitudinal studies have the advantage of evaluating the same animal throughout life, this type of protocol does not allow other measures to be performed, since euthanasia is only done when the animal reaches adulthood.

In summary, we showed that several brain regions are vulnerable to ETS exposure during the early postnatal period and these effects on ^18^F-FDG uptake were observed even a long time after the last exposure. This study corroborates our previous studies, supporting the hypothesis that exposure to tobacco smoke in a critical period interferes with brain development of mice from late infancy to early adulthood.

## Data Availability Statement

The datasets generated for this study are available on request to the corresponding author.

## Ethics Statement

The animal study was reviewed and approved by the Ethics Committee of the School of Medicine (027/14) and the School of Pharmaceutical Sciences (P446/14), University of São Paulo.

## Author Contributions

LT and TM conceived and designed the project. LT, LS, AS, and TA performed all the experiments related to exposure to tobacco smoke and biomarkers quantification under TM supervision. LT, WT, LP, and PS performed the experiments related to PET/CT image acquisition. WT, CR, and DP performed the image processing and analysis. LT, WT, MP, CR, DP, and TM wrote and edited the manuscript. All authors contributed to the manuscript revision, and read and approved the submitted version of the manuscript.

## Conflict of Interest

The authors declare that the research was conducted in the absence of any commercial or financial relationships that could be construed as a potential conflict of interest.
